# VEGF combined with DAPT promotes tissue regeneration and remodeling in vascular grafts

**DOI:** 10.1093/rb/rbad088

**Published:** 2023-10-13

**Authors:** Tao Yang, Guangxu Li, Xifeng Li, Boyang Wei, Hengxian Su, Wenchao Liu, Shenquan Guo, Nan Yang, Tao Xu, Chuanzhi Duan

**Affiliations:** Neurosurgery Center, Department of Cerebrovascular Surgery, The National Key Clinical Specialty, The Engineering Technology Research Center of Education Ministry of China on Diagnosis and Treatment of Cerebrovascular Disease, Guangdong Provincial Key Laboratory on Brain Function Repair and Regeneration, The Neurosurgery Institute of Guangdong Province, Zhujiang Hospital, Southern Medical University, Guangzhou 510282, China; Department of Neurosurgery, The First Affiliated Hospital of Jinan University, Guangzhou, Guangdong, 510630, China; Neurosurgery Center, Department of Cerebrovascular Surgery, The National Key Clinical Specialty, The Engineering Technology Research Center of Education Ministry of China on Diagnosis and Treatment of Cerebrovascular Disease, Guangdong Provincial Key Laboratory on Brain Function Repair and Regeneration, The Neurosurgery Institute of Guangdong Province, Zhujiang Hospital, Southern Medical University, Guangzhou 510282, China; Neurosurgery Center, Department of Cerebrovascular Surgery, The National Key Clinical Specialty, The Engineering Technology Research Center of Education Ministry of China on Diagnosis and Treatment of Cerebrovascular Disease, Guangdong Provincial Key Laboratory on Brain Function Repair and Regeneration, The Neurosurgery Institute of Guangdong Province, Zhujiang Hospital, Southern Medical University, Guangzhou 510282, China; Neurosurgery Center, Department of Cerebrovascular Surgery, The National Key Clinical Specialty, The Engineering Technology Research Center of Education Ministry of China on Diagnosis and Treatment of Cerebrovascular Disease, Guangdong Provincial Key Laboratory on Brain Function Repair and Regeneration, The Neurosurgery Institute of Guangdong Province, Zhujiang Hospital, Southern Medical University, Guangzhou 510282, China; Neurosurgery Center, Department of Cerebrovascular Surgery, The National Key Clinical Specialty, The Engineering Technology Research Center of Education Ministry of China on Diagnosis and Treatment of Cerebrovascular Disease, Guangdong Provincial Key Laboratory on Brain Function Repair and Regeneration, The Neurosurgery Institute of Guangdong Province, Zhujiang Hospital, Southern Medical University, Guangzhou 510282, China; Neurosurgery Center, Department of Cerebrovascular Surgery, The National Key Clinical Specialty, The Engineering Technology Research Center of Education Ministry of China on Diagnosis and Treatment of Cerebrovascular Disease, Guangdong Provincial Key Laboratory on Brain Function Repair and Regeneration, The Neurosurgery Institute of Guangdong Province, Zhujiang Hospital, Southern Medical University, Guangzhou 510282, China; Neurosurgery Center, Department of Cerebrovascular Surgery, The National Key Clinical Specialty, The Engineering Technology Research Center of Education Ministry of China on Diagnosis and Treatment of Cerebrovascular Disease, Guangdong Provincial Key Laboratory on Brain Function Repair and Regeneration, The Neurosurgery Institute of Guangdong Province, Zhujiang Hospital, Southern Medical University, Guangzhou 510282, China; Department of Bio-intelligent Manufacturing and Living Matter Bioprinting Center, Research Institute of Tsinghua University in Shenzhen, Tsinghua University, Shenzhen 518057, People’s Republic of China; Department of Tsinghua Shenzhen International Graduate School, Tsinghua University, Shenzhen 518055, People’s Republic of China; Neurosurgery Center, Department of Cerebrovascular Surgery, The National Key Clinical Specialty, The Engineering Technology Research Center of Education Ministry of China on Diagnosis and Treatment of Cerebrovascular Disease, Guangdong Provincial Key Laboratory on Brain Function Repair and Regeneration, The Neurosurgery Institute of Guangdong Province, Zhujiang Hospital, Southern Medical University, Guangzhou 510282, China

**Keywords:** VEGF, Notch, graft, regeneration, remodeling

## Abstract

Previous research on tissue-engineered blood vessels (TEBVs) has mainly focused on the intima or adventitia unilaterally, neglecting the equal importance of both layers. Meanwhile, the efficacy of grafts modified with vascular endothelial growth factor (VEGF) merely has been limited. Here, we developed a small-diameter graft that can gradually release VEGF and γ secretase inhibitor IX (DAPT) to enhance tissue regeneration and remodeling in both the intima and adventitia. *In vitro*, experiments revealed that the combination of VEGF and DAPT had superior pro-proliferation and pro-migration effects on endothelial cells. *In vivo*, the sustained release of VEGF and DAPT from the grafts resulted in improved regeneration and remodeling. Specifically, in the intima, faster endothelialization and regeneration of smooth muscle cells led to higher patency rates and better remodeling. In the adventitia, a higher density of neovascularization, M2 macrophages and fibroblasts promoted cellular ingrowth and replacement of the implant with autologous neo-tissue. Furthermore, western blot analysis confirmed that the regenerated ECs were functional and the effect of DAPT was associated with increased expression of vascular endothelial growth factor receptor 2. Our study demonstrated that the sustained release of VEGF and DAPT from the graft can effectively promote tissue regeneration and remodeling in both the intima and adventitia. This development has the potential to significantly accelerate the clinical application of small-diameter TEBVs.

## Graphical abstract

**Figure:**
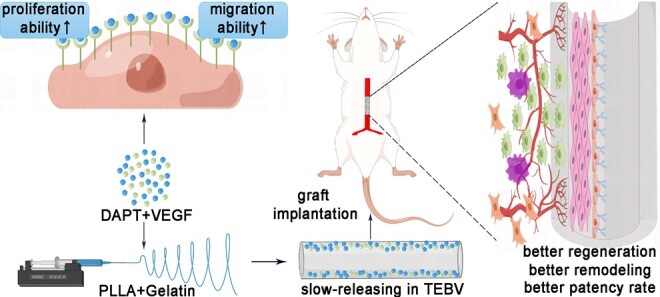

## Introduction

Vascular diseases have extremely high disability and mortality rates, and the number of patients with cardiovascular and cerebrovascular diseases is rising all over the world [1]. Although vascular interventional therapy has been effective in treating numerous vascular diseases [2], vascular bypass remains a crucial therapeutic approach, particularly in cases of severe vessel wall damage [3]. Autologous vessels, including great saphenous veins, brachial arteries and mammary arteries, are still the first choice of vascular bypass grafts because of their high compatibility, high compliance and low antigenicity. However, not all patients have suitable autologous vessels, and these vessels are prone to complications such as aneurysm formation and injury [4]. Consequently, there is a growing demand for tissue-engineered blood vessels (TEBVs). Significant clinical effects have been obtained in the large-diameter vascular graft (Ф > 6 mm) [5], but there still have been no small-diameter (Ф < 6 mm) TEBV that can be directly applied clinically. This is due to the poor patency rate and inadequate tissue remodeling speed associated with small-diameter TEBVs [6].

An increasing body of research has demonstrated the critical importance of rapid endothelialization within the lumen of a graft to prevent blockage [7]. Meanwhile, the adventitia and peri-graft tissue are also crucial for the regeneration and remodeling of the graft [8]. Consequently, the design of vascular grafts should be approached holistically, encompassing both the intima and adventitia, as well as the graft material itself.

Vascular endothelial growth factor (VEGF) has long been recognized as one of the most significant factors in promoting angiogenesis [9]. The introduction of VEGF into TEBV accelerated the endothelialization process [10]. Nevertheless, further improvements to the speed and degree of endothelialization following graft implantation are necessary, as more complete and rapid endothelialization can result in lower rates of blockage and higher compliance. With the deepening of the Tip/stalk cell behavior in angiogenesis, more evidence shows that Notch signaling forms a negative feedback loop with VEGF signaling by down-regulating vascular endothelial growth factor receptor 2 (VEGFR2) expression, which, to some extent, down-regulated the pro-proliferative and pro-migratory ability of VEGF in endothelial cells (ECs) [11–14]. Numerous studies have shown that inhibition of Notch activation using γ-secretase inhibitors can enhance various VEGF-induced angiogenesis processes, such as endothelial cell (EC) proliferation and migration [15–17]. Additionally, Notch pathway inhibition can promote the polarization of macrophages toward the M2 state, which possesses tissue remodeling and anti-inflammatory properties [18, 19]. γ secretase inhibitor IX (DAPT) is one of the potent inhibitors of Notch signaling [20].

Electrospinning is a simple and efficient technology for preparing TEBV. By adjusting the pore size, fiber thickness, spinning agent and other parameters, TEBV can be prepared to promote cell adhesion, migration and proliferation [21]. These spinning agents primarily consist of natural and synthetic polymers that can be utilized in various combinations to enhance the biocompatibility and mechanical properties of TEBVs. Compared with the single polymer, the mixed preparation of natural polymer and synthetic polymer can endow TEBV with better biocompatibility and mechanical properties [22]. l-polylactic acid (PLLA) is a synthetic polymer with good mechanical properties and degradability [23]. Gelatin, a natural polymer, has excellent biocompatibility. Due to its composition which is similar to the extracellular matrix, gelatin can promote cell adhesion, migration and proliferation [24]. Therefore, the mixture of PLLA and gelatin was used as the main raw material. VEGF and/or DAPT were incorporated into the mixture to enable sustained release and prepare TEBVs via electrospinning.

Previous studies on TEBV have predominantly emphasized the endothelialization of the graft lumen. It should be noted that the adventitia also plays a crucial role in facilitating the replacement of the graft with autologous tissue. In this study, we verified the effect of VEGF combined with DAPT on the proliferation and migration of human umbilical vein endothelial cells (HUVECs) *in vitro*. Then we implanted the prepared TEBV into the abdominal aorta of rats and regularly observed the vascular patency. Finally, after euthanasia, we removed the graft and observed the degree of tissue remodeling of its intima and adventitia. In consequence, we found that the graft sustained release of VEGF and DAPT had the best effect on tissue remodeling, not only in the lumen but also in the adventitia of the graft. Furthermore, the joint application of VEGF and DAPT also promoted cell infiltration into the fibers of grafts.

## Materials and methods

### Animals

This study was carried out in adherence to the Declaration of Helsinki. Standard rats (300–400 g) were provided by the Experimental Animal Center of Southern Medical University. All procedures were approved by the Southern Medical University Ethics Committee (Guangzhou, Guangdong, China) and were performed in full compliance with the policies of the National Institutes of Health on the care and use of animals. The ethical approval number is LAEC-2021-102.

### Materials

PLLA was purchased from Corbion Company of the Netherlands. Gelatin was purchased from Medprin Biotech, Germany. Hexafluoroisopropanol was provided by the Shanghai Macklin Company. Recombinant Rat VEGF 164 Protein and Recombinant Human VEGF 165 Protein were purchased from R&D Systems, USA. DAPT was purchased from GLPBIO, USA. EdU kit (EdU-488) was purchased from Beyotime Biotechnology, China. Standard rats (300–400 g) were provided by the Experimental Animal Center of Southern Medical University. All procedures were approved by the Ethics Committee of Southern Medical University.

### Preparation and characterization of the graft

#### Graft fabrication

The grafts were prepared by electrospinning using a setup described previously in detail [25]. In brief, PLLA and gelatin were mixed and dissolved in 1,1,1,3,3,3-hexafluoro-2-propanol at the weight ratio of 3:1 to form a 15% (Wt/V) mixture solution. Both VEGF and DAPT were dissolved through dimethyl sulfoxide. Then VEGF was added into the polymer solution to a final concentration of 10 μg/ml (determined by the previous studies [10]) and DAPT was added into the polymer solution to a final concentration of 8 μM (determined by the live and dead staining of HUVECs, see Supplementary Figure S1). The polymer solution was ejected at a rate of 4 ml/h using an adjustable syringe pump through a 23-gauge stainless-steel needle. Ejected filaments are collected through stainless steel spindles (diameter = 1.5 mm; speed = 100 rpm). A needle-to-collector distance of 14 cm and a positive voltage of 16 kV were applied during electrospinning. Stop the equipment until the wall thickness of the graft reaches approximately 250 μm. The obtained grafts were inelastic. The grafts were placed in a vacuum overnight to remove the residual solvent and sterilized by exposure to UV light for 24 h before implantation.

#### Fiber and pore measurements

The morphology of the fabricated grafts was examined using scanning electron microscopy (SEM). After dehydration, the grafts were placed on a holder and sputter coated with gold, then observed its pore and fiber through SEM. Six images were obtained for each sample.

The average pore size and fiber diameter were analyzed by manual measurement and theoretical calculations based on SEM images. Six images were analyzed by ImageJ software. At least 20 fibers or pores were analyzed per SEM image. The size of each pore was the average between the long and short diameters of the fitted ellipse.

Graft porosity was measured through the liquid intrusion method. After weighing the dry weights (*M*_dry_), the grafts were immersed in 100% ethanol overnight. Then gently wiped the excess ethanol and weighed the grafts to get the wet weights (*M*_wet_). Calculated the graft porosity with the following equations: *V*_EtOH_ = (*M*_wet_ − *M*_dry_)/*Q*_EtOH_, *V*_graft_ = *M*_dry_/*Q*_graft_, porosity = *V*_EtOH_/(*V*_EtOH_ + *V*_graft_). (*V*_EtOH_ is the volume of ethanol entrapped in graft pores, *V*_graft_ is the volume of the graft, *Q*_EtOH_ and *Q*_graft_ is the density of the ethanol and graft).

#### Mechanical testing

The tensile test of the grafts was measured by an Instron machine (MTS E43.104, Shenzhen, China). Grafts were cut into segments of 8 mm before infiltrating in phosphate-buffered saline (PBS), then put it on the Instron machine. Running the instrument with the strain rate of 20 mm × min^−1^ until the graft break, recorded the data and drew the stress–strain curves by Origin Software. The tensile strength and elastic modulus were calculated from the recorded data.

The burst pressure was measured using a pressure gauge and a peristaltic pump. After securing the graft to the perfusion instrument, its surface was covered with parafilm to prevent water leakage. The graft was then perfused with water, and when it ruptured and leaked, the pressure gauge reading was recorded.

The static contact angle of grafts was tested through a contact angle meter, Kino SL250 (Boston, MA, USA). Briefly, deionized water (4 μl) was dropped onto a sample of grafts with a surface area of 2 × 2 cm^2^. Take photos at 3.5 and 4.5 s, then analyze them by ImageJ software.

#### Degradation and release kinetics

After initially weighing and recording (*W*_1_) the TEBV of each group, put grafts into PBS which was maintained at a constant temperature of 37°C and changed weekly. The grafts were removed at 1, 3, 6, 9, 12 and 15 weeks. After vacuum drying, it was weighed and recorded again (*W*_2_). Weight loss (%) = (*W*_1_ − *W*_2_)/*W*_1_ × 100%. Weight loss represents material degradation and the curve is drawn by GraphPad Prism Software. Experiments were performed five times in each group.

The release kinetics of the corresponding factors on grafts were detected by the VEGF Elisa kit (R&D Systems, USA) and DAPT Elisa kit (YuanJu Biotechnology, China). Briefly, the grafts of each group were cut into segments of 8 mm, then soaked in PBS (10 ml) which was maintained at a constant temperature of 37°C. The supernatant (50 μl) was taken every 2 days, and the factor content was determined by the Elisa assay.

### Cell proliferation and migration

For cell proliferation assays, HUVECs (SanDiego, CA, USA) cultured in Endothelial Cell Medium 1001 (ScienCell, USA) were seeded in 24-well plates at a seeding density of 1 **×** 10^5^/cm^2^. Media was supplemented with VEGF (final concentration: 10 ng/ml, initially dissolved in dimethyl sulfoxide) and/or DAPT (final concentration: 1.5 μM, initially dissolved in dimethyl sulfoxide). The same amount of dimethyl sulfoxide (DMSO) was added into the control group to ensure a final concentration of 0.02% DMSO in each group. After incubating cells at 37°C, 5% CO_2_ for 48 h, the EdU working solution (10 μM) was added into the media to incubate another 2 h. Subsequently, cells were fixed, permeabilized, and added with a Click reaction solution (500 μl). Finally, the nuclei were stained with Hoechst and observed under the fluorescence microscope. After obtaining the EdU assay images, counted the number of positive cells directly and calculate the proportion of positive cells.

For cell migration analysis, 5 × 10^5^ cells in 100 μl basic media (without serum) were added to the top well of transwell dishes with a pore size of 8 μm, while the bottom well added ECM (containing 10% serum, 30 ng/ml VEGF and/or 3 μM DAPT). The final amount of DMSO in each group was also the same. At last, the cells that migrated towards the bottom well were stained with Crystal violet after 18 h. The number of migrated cells was counted by ImageJ software and analyzed statistically. When performing cell scratch experiments, the straight line scratch was created by a disposable 100 μl pipette after incubating HUVECs in 6-well plates for 24 h. Residual floating cells were washed off with PBS, then added the basic media (other components are the same as the EdU assay described above except that there is no serum). The photos of the scratches were taken at 0 h and 24 h, respectively. The width of the scratch was measured by ImageJ software and calculated the migration rate accordingly.

For the functional assessment of the VEGF released from grafts to demonstrate whether they remain active, we soaked the VEGF-loaded graft in PBS to obtain the leaching solution after 10 days. The concentration of VEGF released from the graft was measured by the Elisa kit. The leaching solution of grafts loaded with VEGF, the leaching solution of grafts loaded with nothing, the equal concentration of VEGF factor and PBS were added into the cell culture medium separately between the four groups. Then completed the EdU cell proliferation assay and analyzed the results.

The passage number of HUVEC used in the cell proliferation and migration assays was between 4 and 6. To eliminate the potential interference of endothelial cell growth supplement (ECGS) on experimental results, we only added ECGS to the culture medium when the cells were recovered and stabilized. The culture medium used in the cell proliferation and migration assays did not contain ECGS.

### TEBVs transplantation in rats abdominal artery

The adult male standard rats were anesthetized by intraperitoneal injection of sodium pentobarbital (50 mg/kg). The midline of the abdominal incision was made to expose the infra-renal abdominal aorta. Then cross-clamped the aorta between the renal and inferior mesenteric arteries. A graft was inserted end-to-end into the aorta using a 10-0 monofilament nylon suture. A vascular patency test was performed after transplantation, and the incision was sutured after waiting for 15 min to confirm no blood oozing. All operations were performed under aseptic conditions. After recovery from the surgery, the rats were maintained without postoperative anticoagulation treatment. Animal experiment protocol of this study was carried out in adherence to the Declaration of Helsinki. All procedures were approved by the Southern Medical University Ethics Committee (Guangzhou, Guangdong, China) and were performed in full compliance with the policies of the National Institutes of Health on the care and use of animals.

### Doppler ultrasound examination

VEVO 3100 Imaging System (FUJIFILM VisualSonics) was used to assess the graft patency and blood flow velocity. The rats, under isoflurane-anesthetized, were performed with color Doppler imaging at the postimplantation time intervals of 10, 20, and 30 days. The red colors in the Doppler flow spectrum represent forward flow. Unobstructed grafts showed no interruption of the red ultrasound signal, while blocked grafts showed an interruption signal. Peak blood flow velocity was obtained from the blood flow spectrum in color Doppler imaging. Nine rats in each group accepted the graft implantation into the abdominal aorta. Patency rate = unobstructed graft number/9. Draw the survival curve according to a different time and corresponding patency rate through the GraphPad Prism Software.

### Adventitia observation under stereoscopic microscope

Four weeks after graft implantation, rats were anesthetized with sodium pentobarbital to expose the grafts. The vascular density and autologous neo-tissue were observed and recorded through a stereoscopic microscope (Leica, S9i). Blood vessel tracing was processed by Adobe Photoshop 2020, then the length of blood vessels was analyzed by ImageJ software.

### Histological analysis

At the endpoint (one month) of animal evaluation, all rats were sacrificed by pentobarbital injection. Grafts were separated and washed by PBS, then fixed with 2.5% glutaraldehyde or 4% paraformaldehyde overnight, used for SEM or tissue section staining observation, respectively.

For SEM observation, after dehydration of the fixed tissues through a gradient of ethanol, the samples were sputter coated with gold and observed by SEM. The coverage area of ECs in the middle part of the graft was shown in the image taken by SEM. The surface of the lumen covered with ECs is flaky, while graft fibers can be seen in the uncovered area. Use the Adobe Photoshop 2020 software to circle the area covered by ECs in the SEM image and measured it by ImageJ software. The endothelialization rate (%) = cell coverage area/total area.

To evaluate tissue engineering blood vessel permeability, we injected 0.5% Evans Blue dye (2 ml/kg; Solarbio, China) into the tail vein of rats 2 months after graft implantation. Rats were sacrificed 1 h after Evans Blue injection, then the grafts were removed and prepared into a frozen tissue section. After staining the ECs layer of the graft lumen with CD31, the section was observed under a fluorescence microscope. CD31 was characterized under the FITC channel and the content of Evans Blue dye was observed under the Cy5 channel.

For sectioning and staining, the fixed tissues were dehydrated with 30% sucrose solution until the grafts sank to the bottom three times, and changed the sucrose solution during the period. Then the tissues were embedded with optimal cutting temperature compound and flash-frozen with liquid nitrogen, 6 μm cryostat sections were obtained through a cryostat microtome (Leica, CM1905) subsequently. Finally, tissue sections were stained with hematoxylin & eosin (H&E) or immunofluorescence according to routine practice.

ECs were stained using mouse anti-CD31 primary antibody (1:600, Servicebio). Smooth muscle cells (SMCs) and myofibroblasts were performed by rabbit anti-α-SMA primary antibody (1:800, Servicebio). Fibrinogen was stained through rabbit anti-Fibrinogen gamma chain primary antibody(1:800, Abcam). To visualize macrophages, mouse anti-CD68 primary antibody (1:800, Abcam) was used. To analyze M1 macrophages, mouse anti-iNOS primary antibody (1:800, Abcam) was used. To analyze M2 macrophages, rabbit anti-CD163 primary antibody (1:500, Servicebio) and rabbit anti-CD206 primary antibody (1:800, Abcam) were used. Alexa Fluor 488 donkey anti-rabbit (or mouse) IgG (1:500, Invitrogen) and Alexa Fluor 555 donkey anti-rabbit (or mouse) IgG (1:500, Invitrogen) were used as the secondary antibodies. The sections without incubation with primary antibodies were used as negative controls.

Immunofluorescence images were all captured from randomly microscopic fields and analyzed quantitatively using ImageJ software. Then the fluorescence quantitative data are statistically analyzed.

### Western blot

Grafts and their adjacent abdominal aorta were separated at the endpoint of the rats observation. Protein extract and western blot analysis were performed as described previously [26]. Blots were incubated with primary antibodies for VEGFR2 (1:600, Servicebio) and β-actin (1:1000, Servicebio).

Grafts peeling off the adventitia were also carried out in western blot analysis. Blots were incubated with primary antibodies for endothelial nitric oxide synthase (eNOS) (1:1000, Abcam) and β-actin (1:1000, Servicebio).

The grayscale of indicated protein was quantified by ImageJ software.

### Statistical analysis

GraphPad Prism Software Version 9.0 (San Diego, CA, USA) was used for statistical analysis. Multiple comparisons were performed using a one-way ANOVA and two-way ANOVA. Data are expressed as mean ± SEM. *P* < 0.05 was defined to be significant. *P < 0.05, **P < 0.01, ***P < 0.001. All operations, including the establishment of animal models, the monitoring of patency by ultrasound, the staining of tissue sections, the quantitative analysis of data and so on are all carried out by different experimental staff in blinding principles.

## Results

### Grafts fabrication and characterization

**Figure 1.**
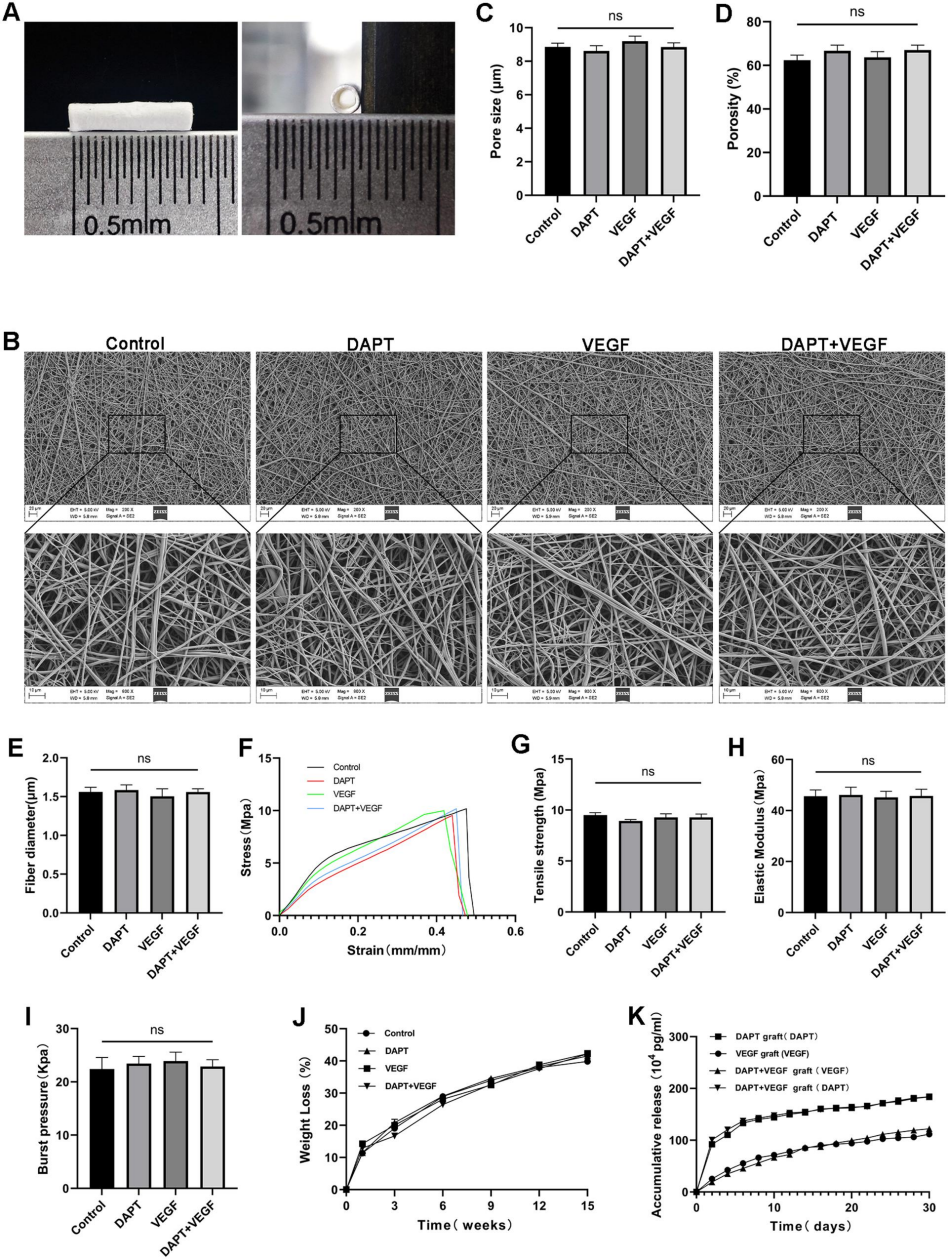
Characterization of grafts. (**A**) Appearance of fabricated grafts. (**B**) Representative SEM images of the inner surface of the grafts. A magnified image of the selected area is shown below. (**C**) Quantitative analysis of pore size from SEM. Pore size was the average between the long and short diameters of the fitted ellipse which is measured by ImageJ software. (**D**) Quantitative analysis of porosity. (**E**) Quantitative analysis of fiber diameter from SEM. The fiber diameter is measured by ImageJ software. (**F**) Stress–strain curves of the grafts. (**G**) Quantitative analysis of tensile strength. (**H**) Quantitative analysis of elastic modulus. (**I**) Quantitative analysis of burst pressure. (**J**) The weight loss curve of the grafts. Weight loss represents material degradation. (**K**) Accumulative release curve of factors from the grafts. Data are represented as mean±SEM (*n* = 3–6/group).

Four groups of TEBVs, which are Control group (adding nothing), DAPT group (adding DAPT), VEGF group (adding VEGF) and DAPT+VEGF group (adding both), were prepared by mixing with different factors in the process of electrospinning. All grafts had an inner diameter of 1.3 mm, an outer diameter of 1.8 mm, and a length of 8 mm (Figure 1A). The fiber and pore morphology and dimension were characterized through SEM. The fibers in each group exhibited a smooth surface and well-defined morphology, and there was no statistical difference in fiber diameter among groups. The averaged diameter was approximately 1.561 ± 0.149 μm for Control group, 1.585 ± 0.161 μm for DAPT group, 1.504 ± 0.238 μm for VEGF group and 1.560 ± 0.102 μm for DAPT + VEGF group (Figure 1B and E). Moreover, the pore size and porosity did not differ significantly among the groups (Figure 1C and D).

The stress–strain curves of each group showed similar trends. The maximum tensile strength of the graft was 9.508 ± 0.406 MPa for Control group, 8.934 ± 0.227 MPa for DAPT group, 9.281 ± 0.602 MPa for VEGF group and 9.283 ± 0.564 MPa for DAPT+VEGF group. The elastic modulus of the grafts all exceeded 40 MPa, which is significantly higher than that of native blood vessels (0.3–1.5 MPa). This indicates that the elasticity of the grafts themselves is not as good as that of natural blood vessels. And the maximum liquid pressure that the grafts can withstand exceeds 20 kPa, so the grafts can withstand the blood pressure impact after being implanted *in vivo* (130 mmHg corresponds to 17.3 kPa). The tensile strength, elastic modulus and burst pressure showed no statistically significant differences among groups (Figure 1F–I). Additionally, the contact angle data for all grafts also did not exhibit any statistically significant differences (Supplementary Figure S2).

The results of the degradation experiment indicate that the addition of various factors did not significantly affect the degradation rate of the grafts. The degradation rate of the grafts was observed to be faster during the initial 3 weeks, followed by a decrease and eventual stabilization (Figure 1J). The factor release curve obtained by the Elisa kit indicated that a significant amount of factor was burst released within the first six days followed by a slow and sustained release. The release characteristics of the dual-delivery system incorporating both VEGF and DAPT were found to be similar to those of the separate delivery systems (Figure 1K).

### Effect of VEGF combined with DAPT on HUVECs function

The result, that there was no significant difference in the ability of HUVECs proliferation and migration between the medium with 0.02% concentration of DMSO and without DMSO, was verified at the beginning of the *in vitro* study (Supplementary Figure S3). Concurrently, to mitigate the potential impact of DMSO solvent on cellular functionality, standardized protocols were implemented whereby both the control and experimental groups were exposed to equivalent final concentrations of DMSO (0.02%).

**Figure 2.**
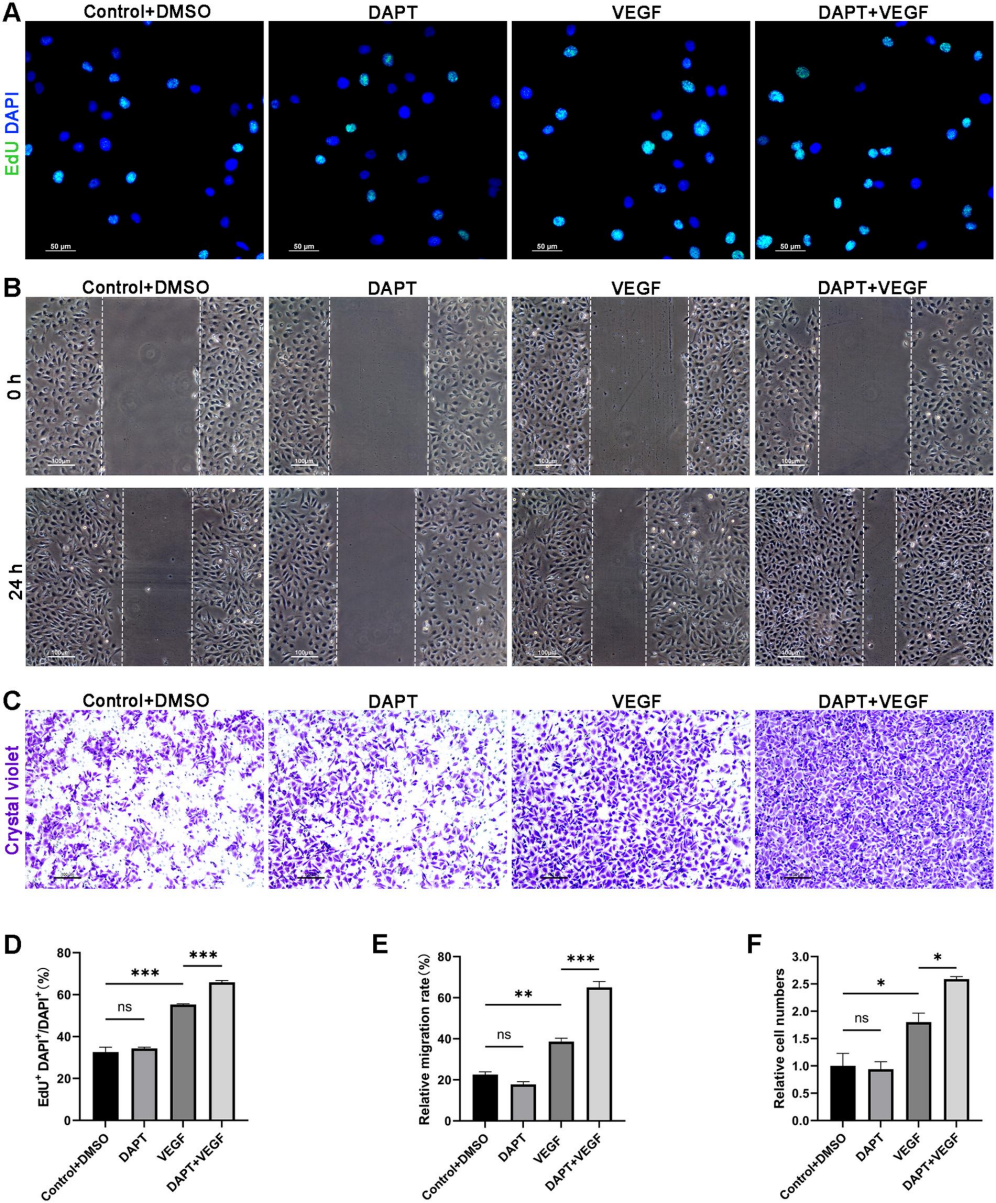
DAPT+VEGF group has the most significant effect on proliferation and migration of HUVECs *in vitro*. (**A**) Representative images of EdU assay of HUVECs. Nuclei are stained blue and EdU+ nuclei are stained green. Cells with more than 50% of their nuclear surface covered in light blue are defined as positive. (**B**) Representative images of wound healing assay of HUVECs. The scratch was imaged with a Leica DMIL microscope and the width of the scratch was measured through ImageJ software. (**C**) Representative images of Transwell assay of HUVECs. Transwell chambers were imaged by a brightfield Leica microscope and the cell numbers were counted in randomly selected fields by ImageJ software. (**D–F**) Quantification analysis of data in (A), (B), and (C). Data are represented as mean±SEM (*n* = 3–4/group). *P < 0.05, **P < 0.01, ***P < 0.001.

EdU cell proliferation assays were performed to assess the effect of VEFG combined with DAPT on HUVECs proliferation. As shown in Figure 2A and D, EdU^+^ cell density was higher upon VEGF group, as compared with those cells cultured in control group and DAPT group. There was no statistical difference between the control group and the DAPT group. These findings suggest that VEGF, a classic growth factor, has significant pro-proliferation ability while using DAPT alone did not alter ECs proliferation. However, when DAPT was used in combination with VEGF, an increase in the density of EdU+ cells was observed, although the increase was limited, it still showed statistical significance. These results suggest that DAPT has a magnifying effect on the pro-proliferation activity of VEGF.

In addition to HUVECs proliferation, we also assessed ECs migration, another event critical for angiogenesis. In Transwell migration assay, unlike adding DAPT alone, adding VEGF alone increases the number of cells migrating toward the bottom. Meanwhile, with the addition of DAPT into VEGF, the number of migrating cells increased remarkably compared with VEGF group, indicating that the pro-migration ability of VEGF was further enhanced (Figure 2C and F). This view was also supported by an EC scratch assay (Figure 2B and E).

The results of VEGF activity shown by EdU assay revealed that the effect of the VEGF released from the grafts on cell proliferation was the same as that of direct use of VEGF factors on HUVECs. Both of them can significantly promote the proliferation of ECs. Importantly, statistical analysis revealed no significant difference between the PBS control and the leaching solution from the unmodified grafts, indicating that the released VEGF factors from the grafts retained their bioactivity without significant loss (Supplementary Figure S4). DAPT is not a bioactive factor, and it has no inactivation problem.

Taken together, these results suggest that the combination of VEGF and DAPT can further promote the pro-proliferation capacity and pro-migration capacity of VEGF. Notably, DAPT alone did not affect the migration and proliferation of ECs.

### Patency of the implanted vascular grafts

**Figure 3.**
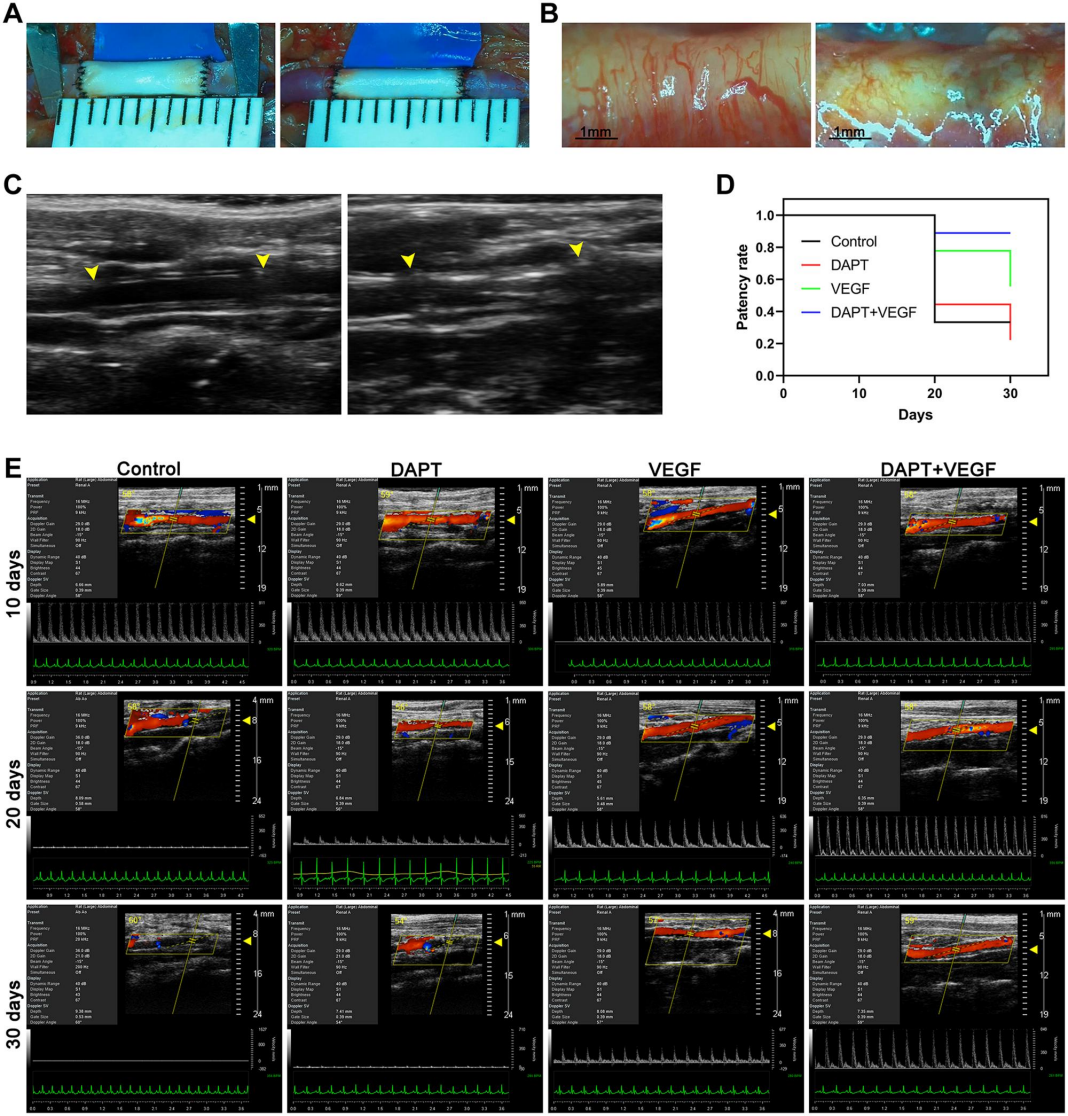
Implantation and regular ultrasound monitoring of the graft. (**A**) Representative images of grafts before and after arterial clamp loosening in DAPT + VEGF group. Images were taken with a Leica stereopsis microscope after completion of anastomosis. (**B**) The difference in adventitia between unoccluded graft (from DAPT + VEGF group) and occluded graft (from control group). Images were taken by a Leica stereo microscope after anesthetizing the rats and exposing the graft. (**C**) The difference in sonographic images between unoccluded graft (from DAPT + VEGF group) and occluded graft (from control group). the ultrasound section is the longitudinal axis of the blood vessel and the ultrasound site is the abdominal aorta. Anastomotic site is indicated by yellow arrows. (**D**) Statistical analysis of graft patency rates (*n* = 9 rats/group). Survival curves were drawn based on the different patency rates for each group at different time points. (**E**) Representative color Doppler ultrasound blood flow images of grafts in four groups at three postimplantation time intervals of 10, 20 and 30 days. Interruption of the red blood flow signal indicates blockage of the graft lumen. The blood flow spectrum is located below the color Doppler ultrasound image. All ultrasound images were taken after rats were anesthetized and the ultrasound section is the longitudinal axis of the blood vessel.

Long-term patency is a critical concern for small-diameter vascular grafts. Our sonographic data revealed that the DAPT + VEGF group exhibited the most favorable graft patency, indicating that the sustained release of VEGF and DAPT from the grafts conferred excellent antithrombotic properties. The patency of the grafts was checked as soon as possible after the implantation through the vascular patency test to ensure a successful anastomosis of the graft (Figure 3A). Based on observations and records made using a stereomicroscope, significant morphological differences were found in the adventitia between unobstructed grafts and obstructed grafts. The adventitia of the unobstructed grafts was clear and translucent with distinct neovascularization. By contrast, the obstructed grafts were obviously deformed, and their adventitia discolored, showing inflammatory infiltration (Figure 3B).

The small-animal ultrasound machine functions in a basic mode wherein grafts and autologous arteries can be distinguished based on differences in tissue elasticity. Meanwhile, significant imaging differences can also be observed between patent grafts and obstructed grafts in the basic mode of the ultrasound. The echo intensity of unobstructed grafts inner wall was clear and uniform, which is similar to the normal artery. However, the echo intensity of occluded grafts lumen was mixed with a visible blockage (Figure 3C). The patency of the graft is monitored every 10 days after implantation. The color Doppler and blood flow spectrum analysis revealed that there were no blockages observed in any of the groups during the initial 10-day period after graft implantation. However, after 20 days, the control group exhibited numerous blocked grafts. Additionally, apart from the DAPT + VEGF group, a considerable decrease in peak blood flow (displayed in the waveform of the color Doppler image) was observed in many unobstructed grafts in each group, indicating partial occlusion. By day 30, only the DAPT + VEGF group maintained a high rate of graft patency, with no significant decrease in peak blood flow observed in the unobstructed grafts. At this stage, although there were still some unobstructed grafts in the VEGF group, their number was lower compared to the DAPT + VEGF group. Furthermore, the peak blood flow in the unobstructed grafts of the VEGF group significantly decreased, suggesting the presence of partial obstruction (Figure 3D and E).

In summary, during our observation period, the grafts sustained releasing VEGF and DAPT can significantly enhance the patency rate in comparison with other groups. These results imply that the endothelialization in the graft of DAPT + VEGF group could be rapidly formed within a short time after implantation.

### Regenerated neotissue in vascular grafts

**Figure 4.**
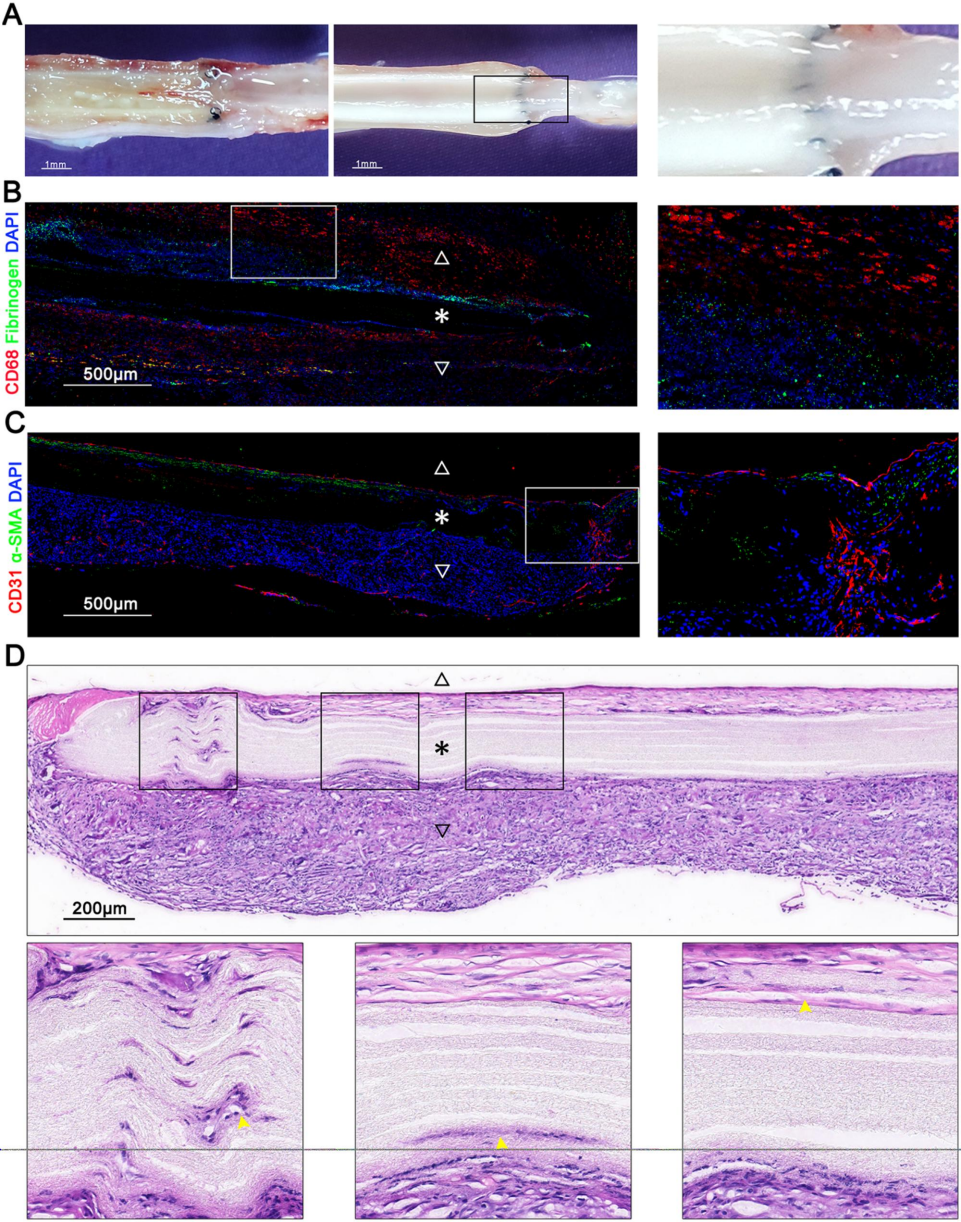
Regenerated neotissue in grafts of DAPT + VEGF group. (**A**) The difference in the luminal surface between occluded graft and unoccluded graft. A magnified image of the selected area is shown on the right. The grafts were cut longitudinally along the long axis and the images were taken after exposing the lumen. (**B**) Immunostaining using indicated antibodies was used to observe thrombus and macrophage infiltration in occluded graft. A magnified image of the selected area is shown on the right. (**C**) Immunostaining using indicated antibodies showed the regeneration of ECs and SMCs in grafts. A magnified image of the selected area is shown on the right. (**D**) H&E staining showed cellular infiltration into the graft fibers. Magnified images of the selected areas are shown below. Graft lumen is indicated by △, graft is indicated by * and adventitia is indicated by ▽. Cellular infiltration is indicated by a yellow arrow.

One month post-implantation, regenerated neotissue, particularly new nutrient vessels, were observed in the adventitia of the graft (Figure 3B). In order to further evaluate the regeneration and remodeling of the transplant, the grafts were extracted from rats under anesthesia and longitudinally sectioned. Although it is challenging and imprecise to observe the differences in the degree of patent graft regeneration among the groups using macroscopic images, clear differences can be observed in the luminal interior between patent grafts and occluded grafts. In the longitudinal section of the unoccluded graft, the lumen was clear and translucent with a layer of new tissue covering the inner surface of the grafts. Conversely, the longitudinal section of occluded grafts revealed thrombosis as the cause of graft occlusion. Notably, in macroscopic images, the DAPT + VEGF group exhibited more pronounced intimal neotissue, with complete coverage of the anastomotic suture by neointima. In contrast, although neointima formation was also observed in patent grafts of other groups, the anastomotic suture was still clearly visible, indicating that the degree of neointima formation was far less than that of the DAPT + VEGF group. Besides, no significant differences were observed in the occluded grafts among the groups (Figure 4A and Supplementary Figure S5A).

Immunofluorescence staining showed that the lumen of the occluded grafts was filled with fibrinogen and macrophages, and the adventitia also had abundant macrophages which indicated an obvious inflammatory infiltration (Figure 4B). On the contrary, unobstructed grafts had neointima containing ECs layer and smooth muscle cells (SMCs) layer and neoadventitia containing rich nourishing vessels. The ECs layer of the grafts in DAPT + VEGF group is closely connected and has good continuity, even at the anastomotic site (Figure 4C). This result was also supported by HE staining of the grafts (Figure 4D and Supplementary Figure S5B).

### Endothelialization and SMCs regeneration of the graft

**Figure 5.**
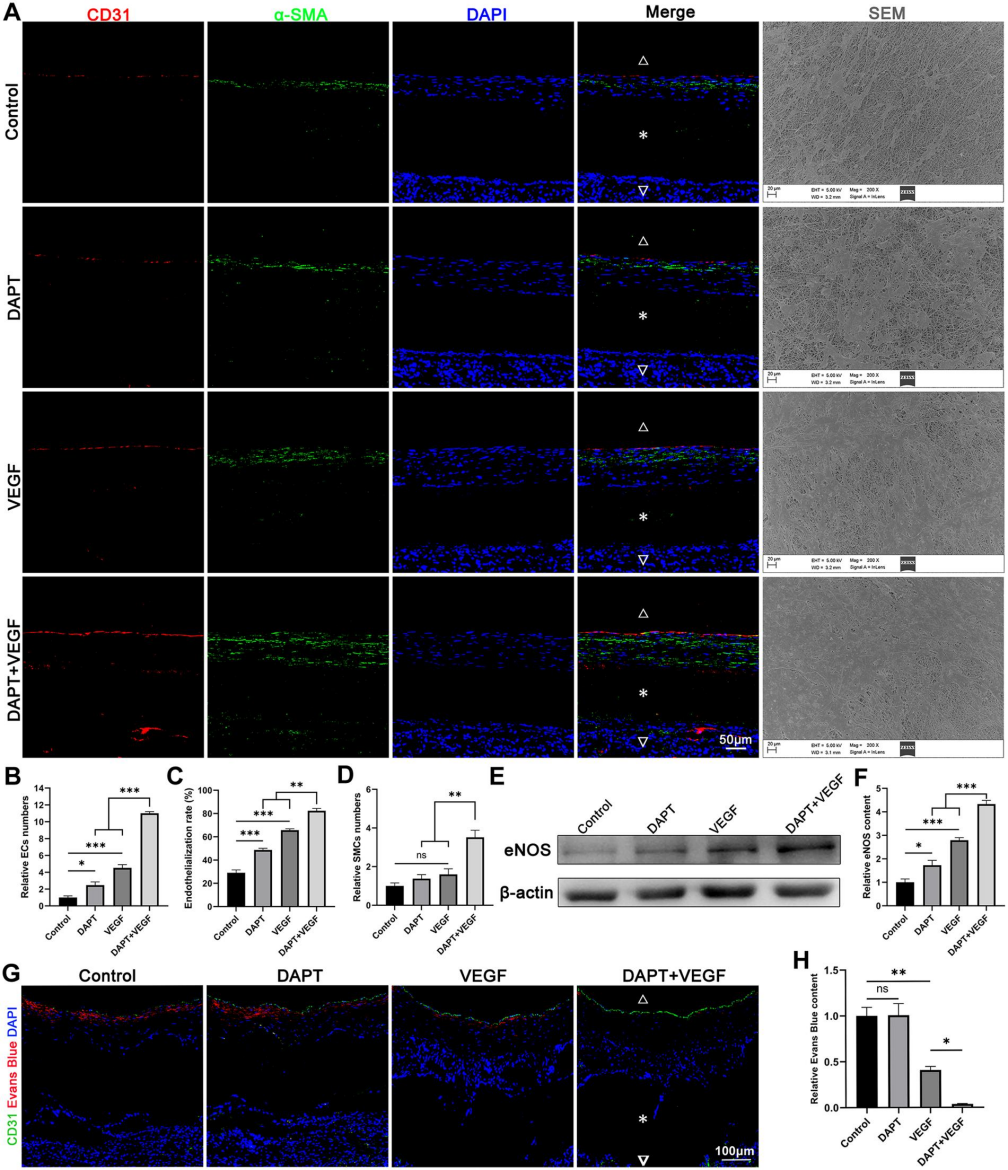
DAPT + VEGF group has the best effect on intimal regeneration. (**A**) Endothelialization and SMCs regeneration were shown in the grafts immunostained by CD31 and α-SMA. Representative SEM images showed the endothelialization process of the grafts after implantation. Images were all taken from the Middle of the grafts. (**B–D**) Quantification of data in (A). Fluorescence intensity and quantification were analyzed by ImageJ software. The EC coverage area in the SEM images is also calculated by ImageJ software. (**E**) Western blot analysis of eNOS protein. (**F**) Quantification of data in (E). The grayscale of indicated protein was quantified by ImageJ software. (**G**) Vascular permeability analysis of the graft. Images were all taken from the Middle of the grafts. Red fluorescence represents the extravasation of Evans Blue dye. Lower permeability represents higher endothelial integrity. (**H**) Quantification of data in (G). Fluorescence intensity and quantification were analyzed by ImageJ software. Graft lumen is indicated by △, graft is indicated by * and adventitia is indicated by ▽. Data are represented as mean±SEM (*n* = 4–5/group). *P < 0.05, **P < 0.01, ***P < 0.001.

Two typical markers of VECs (CD31) and VSMCs (α-SMA) were positively stained and observed at the intima of the middle segment of the grafts. The results indicated that all treatment factors, as compared to the control group, facilitated the recruitment and remodeling of endogenous ECs, thereby promoting *in situ* endothelialization. Moreover, delivery of an appropriate combination of DAPT and VEGF from grafts led to a better endothelialization than VEGF or DAPT alone. The result of endothelialization of the graft lumen, which was the same as that of immunofluorescence, was also examined by SEM. However, only the grafts slow-releasing VEGF and DAPT significantly promoted the regeneration and proliferation of SMCs (Figure 5A–D).

Protein expression of eNOS was measured to assess the functional properties of regenerated ECs using western blot. The results of western blot and relative protein level indicated that eNOS was successfully expressed in the regenerated intima composed of ECs, and group difference in expression levels was consistent with the intimal ECs numbers detected by immunofluorescence (Figure 5E and F).

In addition to the number of regenerated ECs in lumen, the endothelial monolayer integrity is also an important indicator. To assess this, we measured the permeability of regenerated endothelial monolayers using Evans Blue dye extravasation. Our findings revealed that the DAPT + VEGF group exhibited the least extravasation of dye, indicating superior barrier function (Figure 5G and H).

### Neovascularization in adventitia of grafts

**Figure 6.**
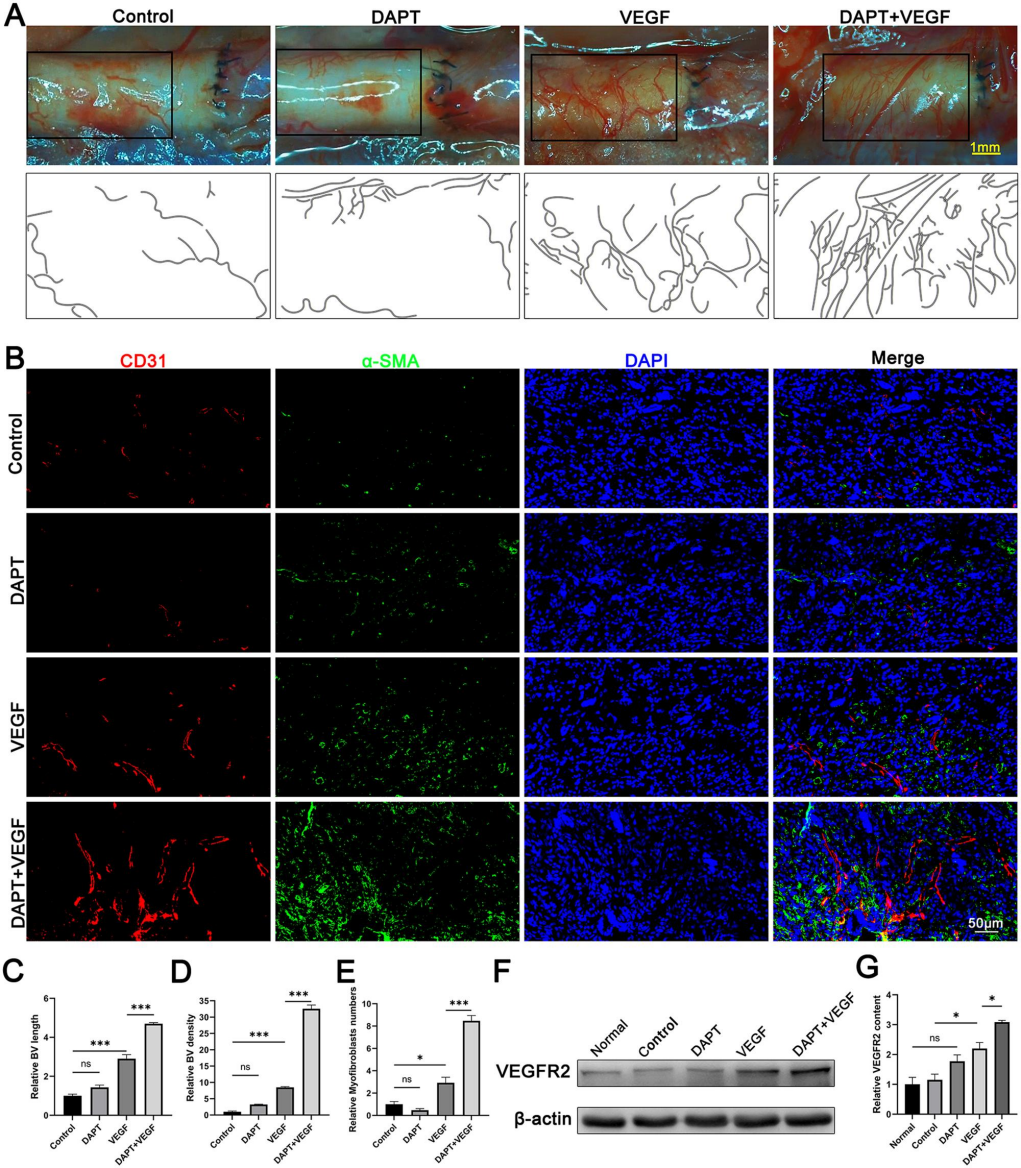
DAPT + VEGF group has the best effect on neovascularization and regeneration of adventitia. (**A**) Representative images of neovascularization in adventitia by stereomicroscope. The graft, native blood vessel and anastomotic end are all shown in the image. After selecting the equal area (5 mm × 3 mm) in the image, Adobe Photoshop 2020 was used to trace the blood vessels, and the blood vessels tracing image of the selected area are shown below. (**B**) Immunostaining using indicated antibodies showed the vascular and myofibroblasts in adventitia. (**C–E**) Quantification of data in (A) and (B). Blood vessel length and fluorescence intensity were analyzed by ImageJ software. (**F**) Western blot analysis of VEGFR2 protein. (**G**) Quantification of data in (F). The grayscale of indicated protein was quantified by ImageJ software. Data are represented as mean±SEM (*n* = 3–4/group). *P < 0.05, ***P < 0.001.

Nutrient vessels are an important aspect of graft remodeling process. Here, we first made a quantitative analysis of the neovascularization in the adventitia of vascular grafts through stereoscopic microscopy. Our findings indicate that VEGF alone is capable of promoting the development of nutrient vessels within the adventitia of grafts, unlike DAPT alone. Furthermore, the adventitia of the grafts that sustained releasing VEGF and DAPT had the highest degree of neovascularization (Figure 6A and C). This view was also supported by an immunofluorescence staining of CD31 in adventitia of grafts (Figure 6B and D).

Besides, immunofluorescence staining showed that compared with VEGF group, VEGF + DAPT group is more effective in promoting the infiltration of myofibroblasts, which are one of the key cells known to be involved in tissue remodeling. No statistically significant differences were observed between the DAPT group and the control group (Figure 6B and E).

### Mechanism of promoting endothelialization and neovascularization

Protein expression of VEGFR2 was measured through western blot. The result showed that the up-regulation of VEGFR2 expression on the surface of ECs was activated in the VEGF group, which was further enhanced in the DAPT + VEGF group. On the contrary, the graft material itself and the addition of DAPT factor alone did not change the expression of VEGFR2 (Figure 6F and G). These results indicated that the further promotion of endothelialization and neovascularization by DAPT in the presence of VEGF may be related to the further increased expression of VEGFR2.

### Cellular infiltration

**Figure 7.**
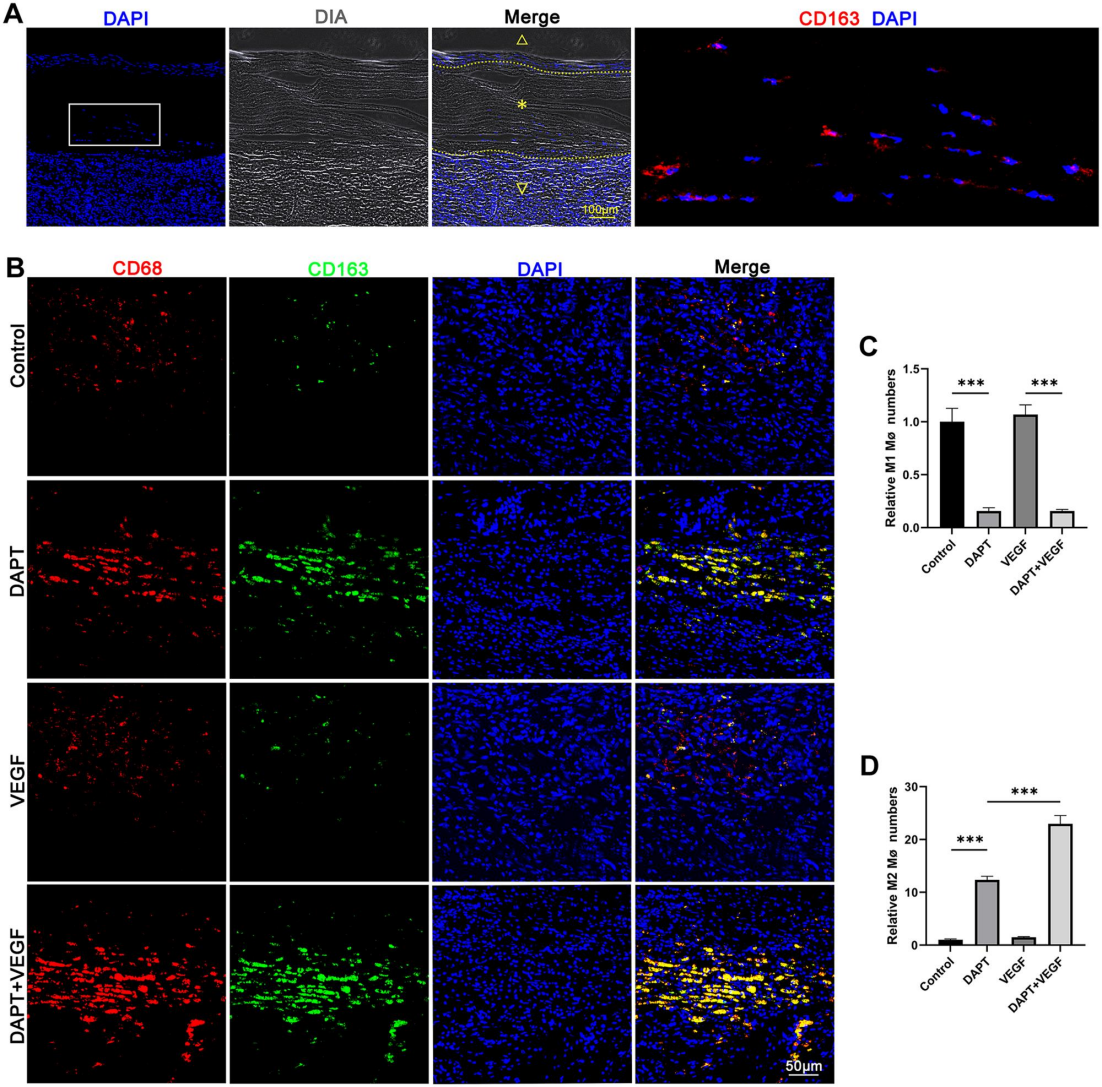
The effect of combined releasing of DAPT and VEGF on macrophage and cellular infiltration. (**A**) Cells infiltrated into the graft fibers were stained by DAPI and a graft outline image was acquired with diascopy (DIA). The selected area, after magnified and immunostained by CD163, is shown on the right. Graft lumen is indicated by △, graft is indicated by * and adventitia is indicated by ▽. The outline of graft is indicated by the dotted line. (**B**) M1 macrophage and M2 macrophage were immunostained by CD68 and CD163 in adventitia of grafts. M1 macrophage is CD68 + CD163- and M2 macrophage is CD68 + CD163+. (**C** and **D**) Quantification of data in (B). The number of M1 and M2 macrophages was counted by ImageJ software. All analyzed images of the graft adventitia are from the middle part of the graft. There are three samples in each group, and each sample takes four regions in the middle part to count and calculate the average value. Data are represented as mean±SEM (*n* = 3/group). ***P < 0.001.

Cell infiltration was detected by H&E and immunofluorescence staining. The section stained by H&E and DAPI showed obvious cell infiltration in the fibers of the graft in DAPT + VEGF group. Not only the cells from the grafts adventitia but also from the grafts intima successfully infiltrated into the fibers of the grafts. The extent of cell infiltration was observed to be more pronounced within the fibers at the anastomosis site, likely due to the effects of external forces during the surgical procedure (Figures 4D and 7A). However, no cells were infiltrated into the graft’s fibers in control group, DAPT group and VEGF group.

For the sake of identification of the infiltrated cell type, the cellular markers including CD68, CD163, CD31 and α-SMA were stained by immunofluorescence. The results showed that the CD163 expression of infiltrating cells was positive, indicating that the cells infiltrated into the graft fibers were mainly M2 macrophages (Figure 7A).

In order to further explore the reason why cell infiltration only occurred in DAPT + VEGF group, M1 and M2 macrophages in the adventitia of grafts were characterized. We performed immunofluorescent staining for pan-macrophage markers (CD68), M1 macrophage markers (iNOS) and M2 macrophage markers (CD163 and CD206). As shown in the immunofluorescence section, DAPT can significantly increase the M2 macrophage numbers and reduce the M1 macrophage numbers in the adventitia of the grafts. Meanwhile, compared with the DAPT group, more M2 macrophages were found in the DAPT + VEGF group. Therefore, the cell infiltration, especially M2 macrophages, in the grafts fibers was thought to be mainly because of the high number and ratio of M2 macrophages in the DAPT + VEGF group (Figure 7B–D and Supplementary Figure S6).

## Discussion

Prior investigations of vascular grafts have typically focused solely on either the intima or adventitia, which is obviously not enough and not comprehensive. To gain a more comprehensive understanding of tissue regeneration and remodeling following graft transplantation, we not only investigated the endothelialization and regeneration of SMCs in the graft lumen but also focused on the macrophage infiltration, myofibroblasts infiltration and nutrient vessel density in grafts adventitia. In addition, we also researched the degree of cell infiltration into the graft fibers.

Cell infiltration is the first step of graft remodeling by autologous tissue. Limited cell infiltration often leads to low rates of regeneration and remodeling progress of rats in the long term [27, 28]. The strategy of relying solely on the degradation of the graft itself to provide space for autologous tissue regeneration has many drawbacks. For example, slow graft degradation can easily lead to calcification, deformation and blockage while fast graft degradation has the risk of bleeding [29, 30]. Therefore, it is particularly important to improve the degree of cell infiltration, because the infiltrating cells can not only accelerate the tissue remodeling process but also fill the gap caused by the graft degradation. The difference in pore size can affect the inward growth of cells. In our experiment, in order to explore whether VEGF combined with DAPT can significantly promote the ability of cell invasion into the graft, a smaller pore size was deliberately designed (about 9 μm) [28]. In addition, it is crucial for us to ensure that the fiber diameter and pore size are consistent across all groups, in order to eliminate any statistical differences in the other properties of the grafts prepared among the groups. We found, except for the VEGF + DAPT group, other groups did not show cells infiltrating into the graft.

Upon implantation of vascular grafts, inflammation serves as a critical physiological response [31–33]. Among various inflammatory cells, macrophages play a crucial role in regulating the balance between pro-inflammatory and anti-inflammatory functions, which is accomplished through the phenotype switching of M1 and M2 [34, 35]. Many studies have shown that excessive M1 macrophage infiltration will lead to graft calcification and fibrosis, while excessive M2 macrophage infiltration will cause better tissue regeneration and remodeling [4, 36].

It has also been reported that inhibition of the Notch signal pathway can promote the polarization of macrophages to M2 phenotypic [18, 19]. Here, we introduced Notch inhibitor into vascular grafts for the first time and studied their effect on macrophages in adventitia of the vascular grafts. Our results indicated that the combined treatment of DAPT and VEGF led to a significant increase in the number of M2 macrophages, which facilitated the successful infiltration of M2 macrophages into the graft fibers. In addition, we also found that the number of myofibroblasts was the largest in the adventitia of grafts in DAPT + VEGF group. Obviously, DAPT combined with VEGF greatly promoted the process of tissue remodeling in the graft.

Arteries are typically composed of three layers: intima, media and adventitia [37]. ECs in intima can effectively prevent thrombosis and secrete a variety of activity regulatory factors, such as NO, endothelin and, so on. The media, which consists of SMCs, plays a crucial role in vasoconstriction, tensile strength, and compliance [38]. Most studies mainly focus on the rapid endothelialization of the vascular graft while ignoring the importance of SMCs. In fact, for the final outcome that the graft is completely replaced by autologous tissue, the regeneration and proliferation of SMCs are equally important [4]. Our study demonstrated that slow-releasing VEGF and DAPT were effective in achieving the best patency in grafts, in part due to the superior endothelialization observed in this group. Beyond that, the joint efforts of VEGF and DAPT can promote the regeneration and proliferation of SMCs, which is an important part of autologous angiogenesis. At the same time, in order to ensure that the regenerated ECs are active and functional, we made a quantitative analysis of eNOS through western blot [38]. As the result shows, the function of proliferated ECs was good in all groups. Besides, vascular permeability results also showed that the DAPT + VEGF group had the highest integrity of ECs and the best barrier function.

The regeneration and remodeling of tissues are inseparable from the nutrition and support of blood vessels. Insufficient vascular density can significantly impede tissue regeneration, leading to fibrosis and scarring [39–41]. The blood vessels in the adventitia of vascular grafts are necessary for tissue regeneration. We quantitatively analyzed the nutrient vessels in the adventitia of vascular grafts for the first time. We found that VEGF combined with DAPT can significantly increase the vascular density of the adventitia, thus giving a better blood supply to cope with the complicated process of tissue regeneration and reconstruction.

Previous research has demonstrated that VEGF can promote the mobilization and upregulation of VEGFR2 in ECs [42–46]. Other studies have also shown that Notch signaling, activated by VEGF, provides negative feedback control of VEGF signaling through down-regulating the expression of VEGFR2 [11–14]. Notch inhibitors can block this negative feedback pathway and increase the expression of VEGFR2, thereby promoting the proliferation and migration of ECs and neovascularization [15–17]. Although VEGF promotes the mobilization and expression of VEGFR2, the Notch negative feedback pathway limits the expression-promoting effect. Nevertheless, the addition of DAPT can attenuate this negative feedback mechanism, thus maximizing the expression of VEGFR2 to enhance the pro-angiogenic ability of VEGF. Our western blot results of VEGFR2 protein are consistent with the above point of view.

Our *in vitro* results are slightly different from *in vivo* results. DAPT alone cannot promote the proliferation and migration of HUVECs, which is different from the result *in vivo*. We posit that the observed disparity between our *in vitro* and *in vivo* results may be attributed to the intricate internal environment of rats. Specifically, the blood’s abundance of growth factors may interact with the slow-releasing DAPT-linked grafts to promote the endothelialization of the lumen. However, this interaction may not be as apparent in the adventitia of the grafts.

## Conclusion

The present study has demonstrated that compared with other groups, the vascular grafts sustained releasing DAPT and VEGF have the best effect on tissue regeneration and remodeling. Specifically, in the intima of the graft, these grafts promote the regeneration and proliferation of endothelial and SMCs. In the adventitia of the graft, they stimulate neovascularization and the polarization of M2 macrophages. Notably, only the DAPT + VEGF group exhibited discernible infiltration into the graft fibers. As such, our study has produced an effective small-diameter engineered blood vessel that exhibits enhanced tissue regeneration and remodeling capabilities in the intima and adventitia of the grafts, as well as in the graft material itself.

## Supplementary Material

rbad088_Supplementary_Data
