# Supplementary material for: VEGF combined with DAPT promotes tissue regeneration and remodeling in vascular grafts
Source: Regen Biomater. 2023 Oct 13;10:rbad088. doi: 10.1093/rb/rbad088 (PMC10603585; doi:10.1093/rb/rbad088)
Supplement: rbad088_Supplementary_Data [file rbad088_supplementary_data.docx]

***Supplementary Material***


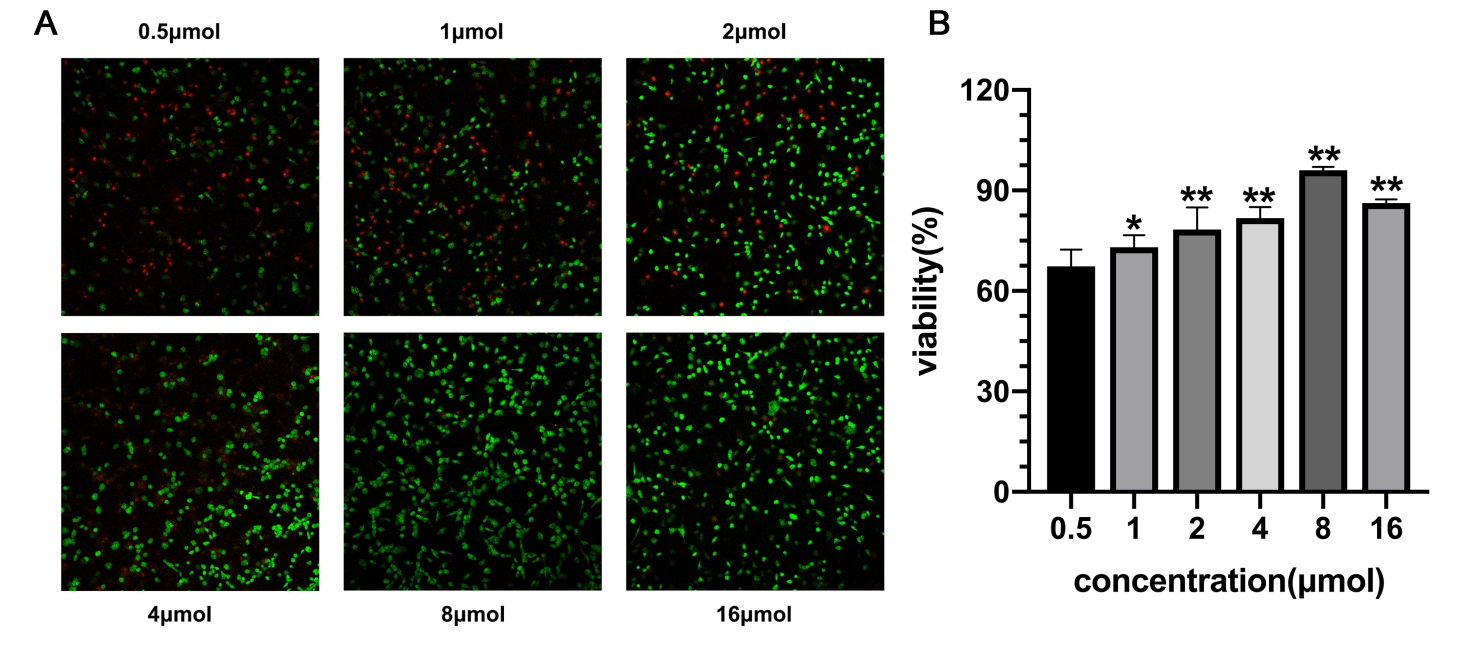


**Fig. S1.** **The best concentration of DAPT, which was determined by the live and dead staining of HUVECs, is 8 μM.** **(A)**HUVECs were co-cultured with grafts prepared with different concentrations of DAPT for 7 days and then stained by live and dead staining. Red fluorescence represents dead cells, while green fluorescence represents living cells **(B)**Quantitative analysis of data in A. The number of living and dead cells was counted by ImageJ software. Data are represented as mean±SD (n=5/group). **^*^**P<0.05, **^**^**P<0.01, **^***^**P<0.001.

**
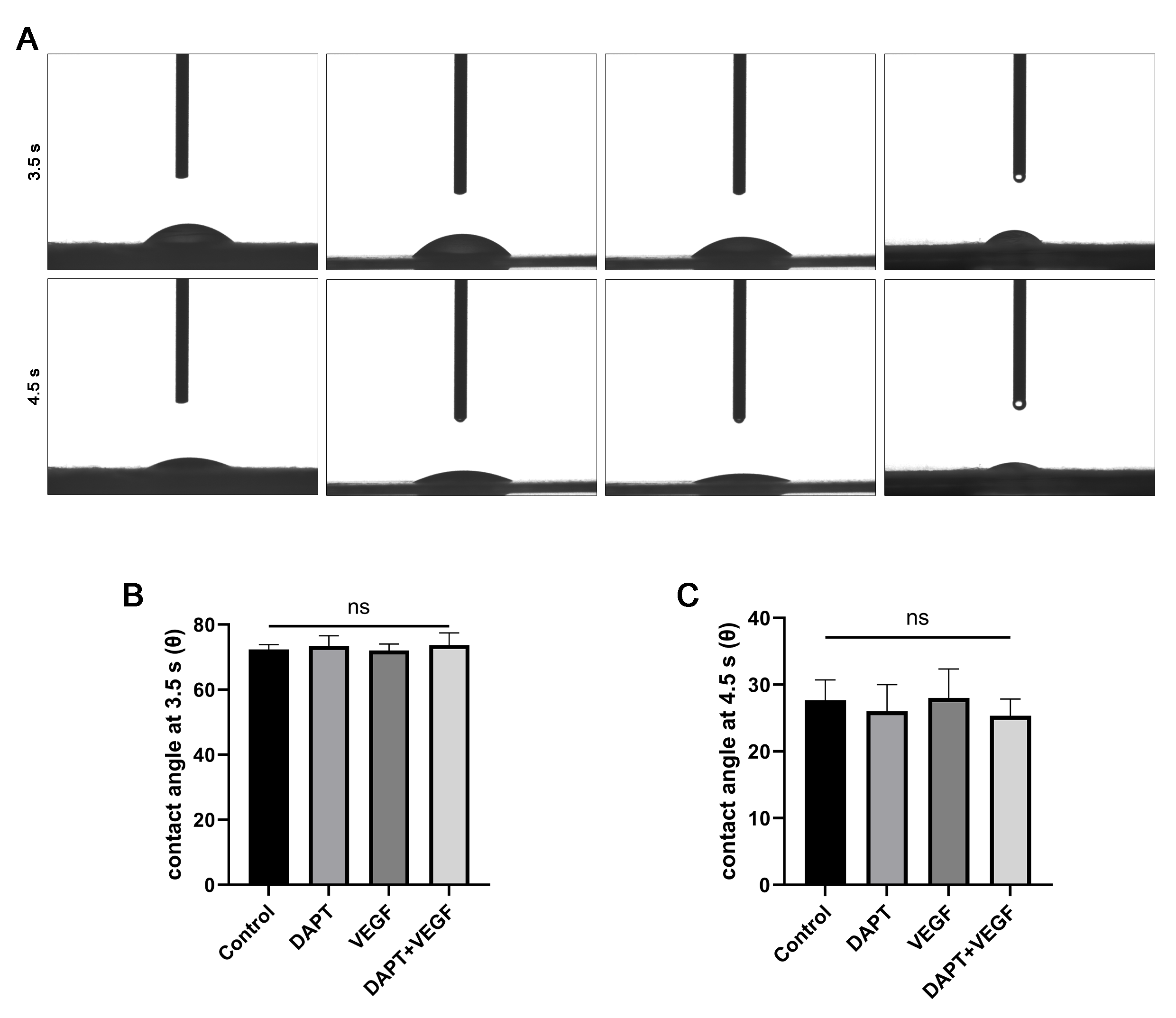
**

**Fig. S2.** **Characterization of hydrophilicity of grafts**. **(A)**The contact angle on the surface of grafts at 3.5 s and 4.5 s. **(B-C)** Quantitative analysis of data in A. Contact angle was measured by ImageJ software. Data are represented as mean±SD (n=3/group).

**
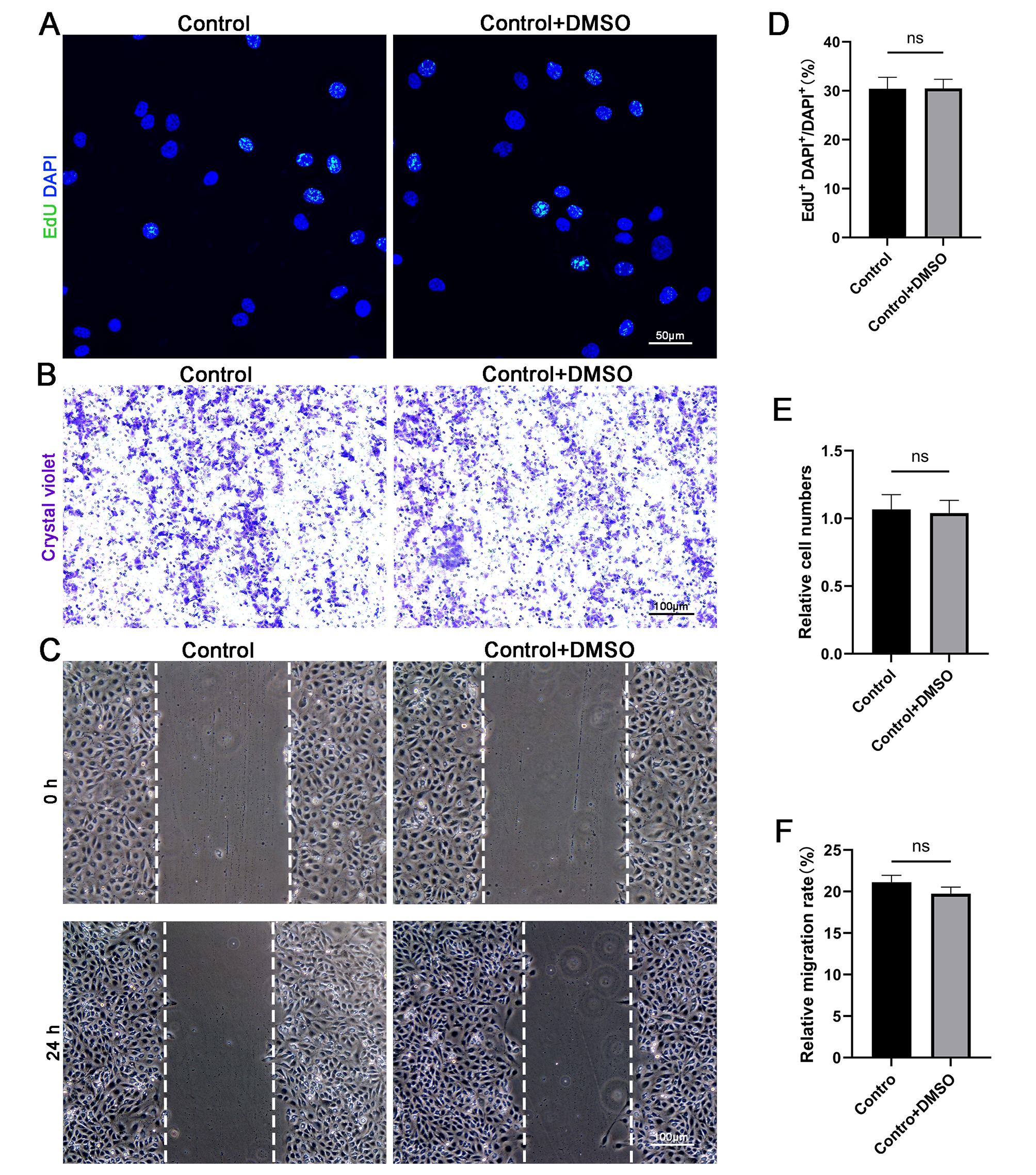
**

**Fig. S3.** **The effect of DMSO(0.02%,v/v) on proliferation and migration of HUVECs (A)**EdU assay showed that DMSO did not affect the proliferation of HUVECs. **(B)**Transwell assay showed that DMSO did not affect the migration of HUVECs. **(C)**Wound healing assay showed that DMSO did not affect the migration of HUVECs. **(D-F)** Quantitative analysis of data in A, B and C. Data are represented as mean±SD (n=3/group).


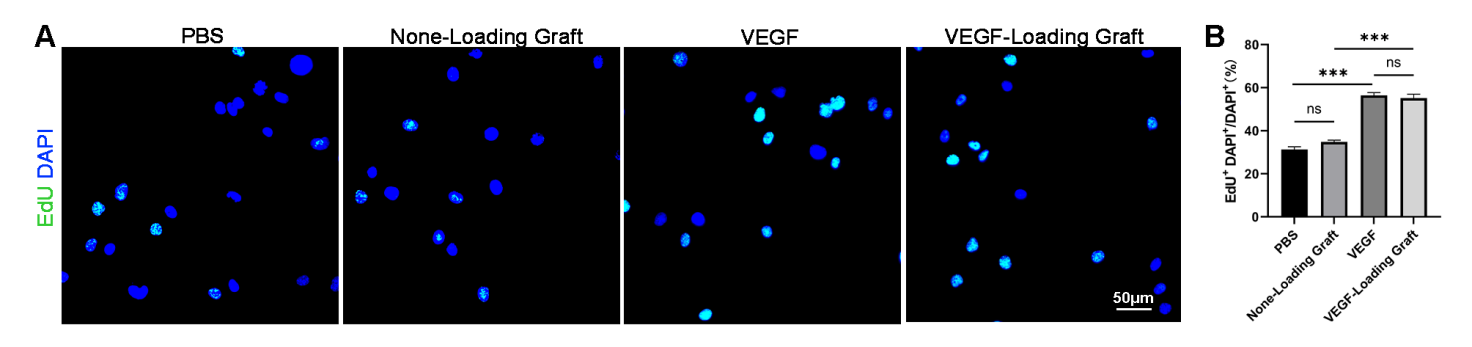


**Fig. S4. Functional assessment of the VEGF released from grafts to demonstrate whether VEGF remains active. (A)**EdU assay showed VEGF activity through its effect on the proliferation ability of HUVECs. **(B)**Quantitative analysis of data in A. Data are represented as mean±SD (n=3/group).


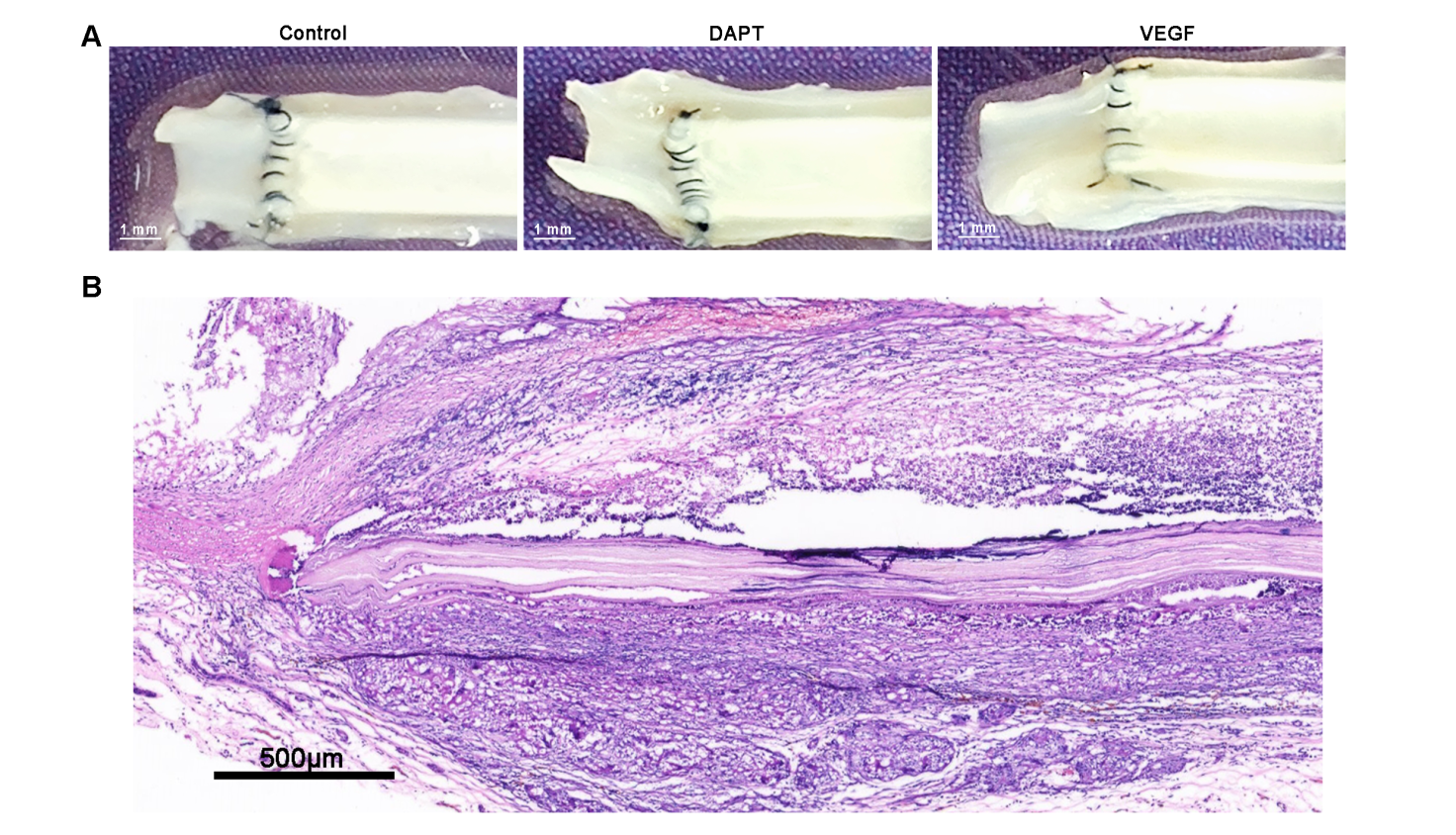


**Fig. S5.** **Regenerated neotissue in grafts of other groups. (A)**The luminal surface of unoccluded graft in other groups. **(B)**Representative image of occluded grafts by H&E staining.

**
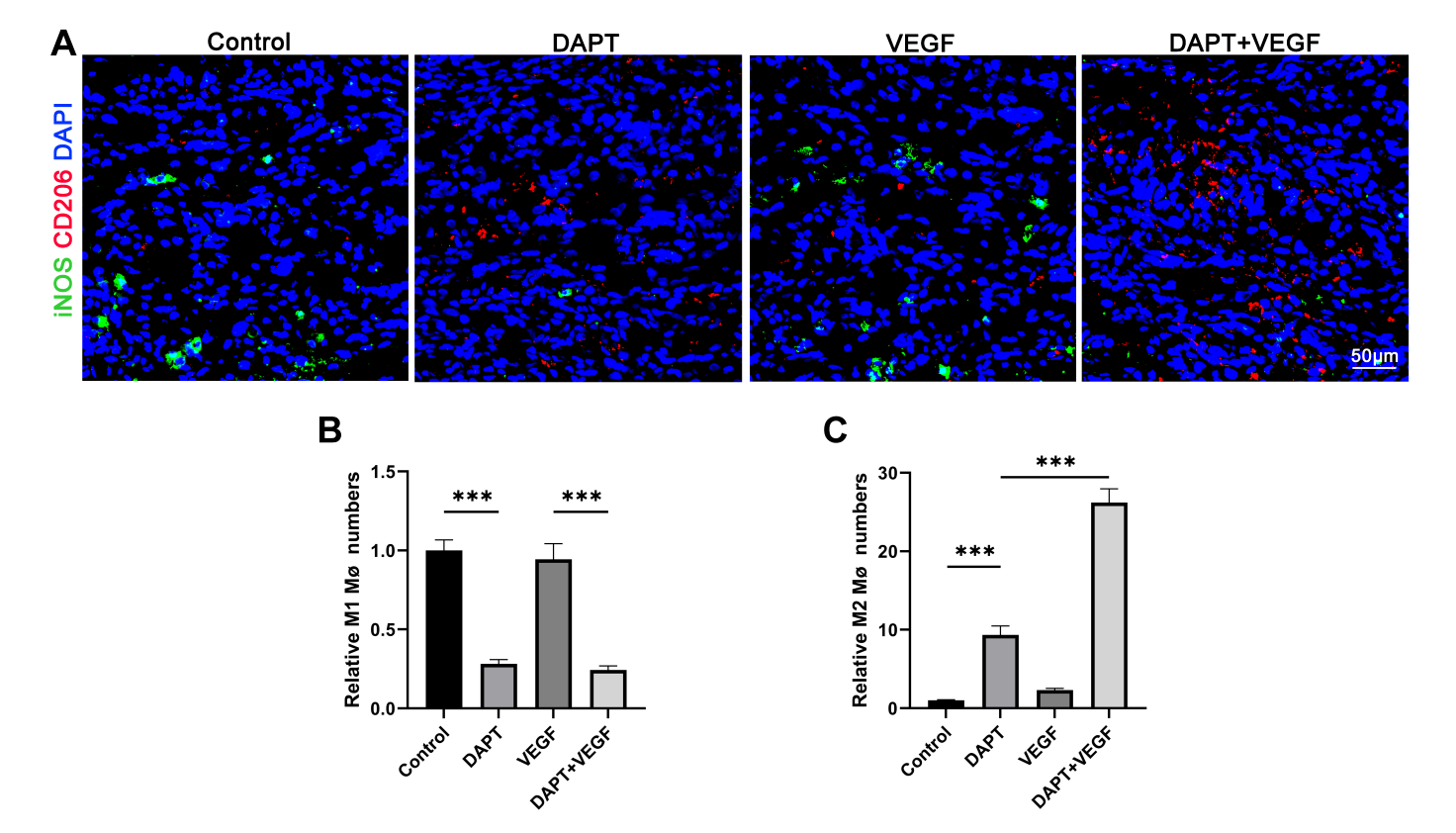
Fig. S6.** **Infiltration of macrophages in the graft adventitia (A)**M1 macrophage and M2 macrophage were immunostained by iNOS and CD206, respectively. **(B-C**)Quantification of data in A. Data are represented as mean±SEM (n=3/group). *P<0.05, **P<0.01, ***P<0.001.
